# Concomitant Enhancement of HIV-1 Replication Potential and Neutralization-Resistance in Concert With Three Adaptive Mutations in Env V1/C2/C4 Domains

**DOI:** 10.3389/fmicb.2019.00002

**Published:** 2019-01-17

**Authors:** Naoya Doi, Masaru Yokoyama, Takaaki Koma, Osamu Kotani, Hironori Sato, Akio Adachi, Masako Nomaguchi

**Affiliations:** ^1^Department of Microbiology, Tokushima University Graduate School of Medical Sciences, Tokushima, Japan; ^2^Laboratory of Viral Genomics, Pathogen Genomics Center, National Institute of Infectious Diseases, Tokyo, Japan; ^3^Department of Microbiology, Kansai Medical University, Osaka, Japan

**Keywords:** HIV-1, Env, adaptive mutation, CD4, replication potential, neutralization sensitivity, Env structure

## Abstract

HIV-1 Env protein functions in the entry process and is the target of neutralizing antibodies. Its intrinsically high mutation rate is certainly one of driving forces for persistence/survival in hosts. For optimal replication in various environments, HIV-1 Env must continue to adapt and evolve through balancing sometimes incompatible function, replication fitness, and neutralization sensitivity. We have previously reported that adapted viruses emerge in repeated and prolonged cultures of cells originally infected with a macaque-tropic HIV-1_NL4-3_ derivative. We have also shown that the adapted viral clones exhibit enhanced growth potentials both in macaque PBMCs and individuals, and that three single-amino acid mutations are present in their Env V1/C2/C4 domains. In this study, we investigated how lab-adapted and highly neutralization-sensitive HIV-1_NL4-3_ adapts its Env to macaque cells with strongly replication-restrictive nature for HIV-1. While a single and two mutations gave a significantly enhanced replication phenotype in a macaque cell line and also in human cell lines that stably express either human CD4 or macaque CD4, the virus simultaneously carrying the three adaptive mutations always grew best. Entry kinetics of parental and triple mutant viruses were similar, whereas the mutant was significantly more readily inhibited for its infectivity by soluble CD4 than parental virus. Furthermore, molecular dynamics simulations of the Env ectodomain (gp120 and gp41 ectodomain) bound with CD4 suggest that the three mutations increase binding affinity of Env for CD4 in solution. Thus, it is quite likely that the affinity for CD4 of the mutant Env is enhanced relative to the parental Env. Neutralization sensitivity of the triple mutant to CD4 binding site antibodies was not significantly different from that of parental virus, whereas the mutant exhibited a considerably higher resistance against neutralization by a CD4-induced epitope antibody and Env trimer-targeting V1/V2 antibodies. These results suggest that the three adaptive mutations cooperatively promote viral growth via increased CD4 affinity, and also that they enhance viral resistance to several neutralization antibodies by changing the Env-trimer conformation. In total, we have verified here an HIV-1 adaptation pathway in host cells and individuals involving Env derived from a lab-adapted and highly neutralization-sensitive clone.

## Introduction

HIV-1 Env protein consists of gp120 and gp41, which are cleaved and matured products of the gp160 precursor protein (Freed and Martin, 1995, 2013; Clapham and McKnight, 2002; Wilen et al., 2012). Gp120 is a surface protein of Env, and contains discontinuous conserved (C1–C5) and variable regions (V1–V5), whereas gp41 has several functional domains including fusion peptide, ectodomain, transmembrane domain, and cytoplasmic tail. HIV-1 Env works as a trimer of a gp120-gp41 heterodimer molecule on viral entry into host target cells. HIV-1 entry process is initiated by attachment of gp120 to cellular receptor CD4. Upon its binding, gp120 experiences structural rearrangements required for interaction with cellular co-receptor, CCR5 and/or CXCR4. Binding of gp120 to the co-receptor triggers large conformational changes of gp41, and thereby induces virus-cell membrane fusion. As such, successful viral entry into cells is achieved by continuous changes in the conformation of gp120 and gp41 proteins, and is critical for efficient viral replication. In addition to functions in the entry process, Env is also targeted by neutralizing antibodies (NAbs), because it is the only viral protein expressed on virion surface. NAbs are categorized by defining an epitope or epitope cluster on Env: CD4 binding site (CD4bs), CD4-induced epitope (CD4i), V1/V2, Glycan-V3, silent face center, fusion peptide, subunit interface, and membrane-proximal external region (Benjelloun et al., 2012; Kwong and Mascola, 2018). To escape binding to NAbs and maintain optimal replication level, HIV-1 Env possesses high ability to mutate and adapt to various surrounding environments. On one hand, mutations in HIV-1 Env can affect both replication capability and neutralization susceptibility: a decrease in replication fitness by a mutation conferring resistance to NAbs; an increase in neutralization sensitivity by a growth-enhancing mutation. Thus, in the adaptation process, it is biologically important for HIV-1 Env to appropriately balance the efficient viral entry via interaction with receptor/co-receptors and the escape from recognition of NAbs by masking epitopes.

Virus adaptation studies have provided a large amount of useful information on viral replicative cycle through extensive analyses of newly emerged mutations in viral genome and their functional and structural effects on viral proteins. For HIV-1, *in vitro* adaptation experiments have revealed various mutants with phenotypes characteristic of viruses resulted from selection pressures, such as antiviral drugs, restriction factors, or limited expression of viral receptor/co-receptors (Trkola et al., 2002; Kuhmann et al., 2004; Pacheco et al., 2008, 2010; Yoshimura et al., 2014; Garg et al., 2016). However, the adaptation of HIV-1 primary isolates to T-cell lines or peripheral blood mononuclear cells (PBMCs) generally and specifically led to better-growing variants with an enhanced sensitivity to soluble CD4 (sCD4) and several NAbs (Moore and Ho, 1995; Wrin et al., 1995; Clapham and McKnight, 2002; Pugach et al., 2004). In addition, while adapting HIV-1 primary isolates to cells with a low CD4 expression (CD4^low^ cells) resulted in an increase in viral CD4-binding ability and in viral infectivity for CD4^low^ target cells including macrophages, these changes were accompanied by a reduced infectivity in CD4^high^ T-cells and by an augmented sensitivity to NAbs (Beauparlant et al., 2017). On one hand, it has been shown that Env proteins from circulating HIV-1 strains have a reduced binding capacity to macaque CD4. An SIV/HIV-1 chimeric virus (SHIV) with a circulating HIV-1 *env* gene showed enhanced macaque CD4-mediated entry following adaptation to macaque cells by acquiring amino acid changes in Env, but its sensitivity to several NAbs was concomitantly increased (Humes and Overbaugh, 2011; Humes et al., 2012; Boyd et al., 2015). Although adaptation pathways seemed to vary depending on virus strains and host environments used in the experiments (van Opijnen et al., 2007), virus affinity to sCD4 and virus sensitivity to several NAbs tend to increase through growth adaptation of primary HIV-1 in cell cultures.

We have previously demonstrated that macaque-tropic HIV-1 derivatives (HIV-1mt), which carry minimal portions of SIVmac_MA239_ genome, can variously and successfully adapt to different macaque cell lines (Kamada et al., 2006; Nomaguchi et al., 2008, 2013a,c, 2014; Yokoyama et al., 2016). This experimental system composed of HIV-1mt clones and macaque cell lines serves for a model study to understand how HIV-1 mutates and adapts to replication-restrictive environments. Our prototype HIV-1mt clone designated ScaVR, which was constructed from a lab-adapted and neutralization-sensitive HIV-1_NL4-3_ strain, replicated poorly in macaque cells (Kamada et al., 2006). In attempts to increase viral replication efficiency, we repeatedly performed prolonged cultivations of macaque cells infected with various HIV-1mt clones. First, we successfully obtained an adapted (growth-enhanced) clone of ScaVR designated NL-DT5R (5R) (Kamada et al., 2006). The 5R genome contained two synonymous mutations [one in long terminal repeat (LTR) and another in *pol*-protease region], and two non-synonymous mutations in *env*. Then, the other adapted viruses were obtained through long-term cultures of macaque cells infected with 5R (Nomaguchi et al., 2008, 2013a). Of several mutations identified in their genomes, only two non-synonymous mutations (one in *pol*-integrase and another in *env* regions) actually promoted viral replication potential. Importantly, the enhancement of viral replication efficiency in macaque cells was indeed reflected in viral growth ability in macaque individuals (Igarashi et al., 2007; Saito et al., 2011). These results indicated that the adaptive mutations identified in cell cultures certainly contribute to promotion of viral growth in individuals. In this work, we studied functional and structural bases for viral growth enhancement by the three Env mutations that spontaneously emerged during repeated *in vitro* adaptation processes. We aimed to clarify how a highly neutralization-sensitive virus clone adapts to a replication-restrictive condition while maintaining viral replication and neutralization-escape potentials.

## Materials and Methods

### Plasmid DNA

A prototype HIV-1mt clone, ScaVR (Kamada et al., 2006), was used as a parental clone in this study. Introduction of mutations into *env* gene was carried out by site-directed mutagenesis with Pfu DNA polymerase (Agilent Technologies). Human CD4 and cynomolgus (CyM) CD4 coding sequences were amplified by PCR using cDNAs synthesized from a human T-cell line H9 and a CyM lymphocyte line HSC-F, respectively. Amplified human- and CyM-CD4 DNA fragments were cloned into the *Not*I site in pCEP4 vector (Thermo Fisher Scientific Inc.), and resultant clones were named H9-CD4+pCEP4 and HSCF-CD4+pCEP4, respectively. Amino acid sequence of CD4 derived from H9 cells was different from that of human CD4 (GenBank accession number AAB51309) at amino acid position 176 (L to F). Amino acid sequence of CD4 from HSC-F cells was identical to that of CyM CD4 (GenBank accession number EHH66018).

### Cells

A human embryonic kidney cell line 293T (Lebkowski et al., 1985) and a reporter cell line, TZM-bl carrying a *luciferase* gene driven by viral LTR (Platt et al., 1998, 2009), were cultured in minimal essential medium (MEM) supplemented with 10% heat-inactivated fetal bovine serum (hiFBS). HSC-F cells (Akari et al., 1999) and a human T-cell line A2.01 (Folks et al., 1986) were cultured in RPMI1640 with 10% hiFBS. A2.01 cells were negative for surface CD4 and CCR5 expression, and positive for surface CXCR4 expression (data not shown). A2.01 cells stably expressing surface human- or CyM-CD4, designated A2.01/HuCD4 and A2.01/CyMCD4, respectively, were generated as follows. A2.01 cells were transfected with H9-CD4+pCEP4 or HSCF-CD4+pCEP4 by using Amaxa Cell Line Nucleofector kit V (Lonza Ltd.) and Nucleofector II device (Lonza Ltd.). Cells proliferated in the presence of 400 μg/mL hygromycin B (Sigma-Aldrich Co. LLC.) were pooled, and used for infection experiments.

### Virus Preparation and Reverse Transcriptase (RT) Assay

Virus stocks were prepared from 293T cells transfected with proviral clones by calcium phosphate co-precipitation method (Adachi et al., 1986) or lipofectamine 2000 (Thermo Fisher Scientific Inc.). Virion-associated RT activity was measured as previously described (Willey et al., 1988; Nomaguchi et al., 2013b).

### Multi-Cycle Infection Assay

Equal RT units (2 × 10^6^) of virus stocks were inoculated into HSC-F cells (2 × 10^5^), and cells were cultured in the presence of 50 units/mL of recombinant human interleukin-2 (IL-2) (Bio-Rad Laboratories Inc.) throughout the observation period. A2.01/HuCD4 and A2.01/CyMCD4 cells (1 × 10^5^) were infected with equal RT units (2 × 10^5^ and 1 × 10^6^, respectively), and cultured in the presence of 400 μg/mL hygromycin B throughout the observation period. Virus replication was monitored by RT activity released into the culture supernatants.

### Single-Cycle Infection, Entry Kinetics Assay, and Neutralization Assay

TZM-bl cells were seeded onto a well of a 96-well plate (4 × 10^3^), and on the next day, equal RT units (2 × 10^3^ ∼ 1 × 10^4^) were inoculated into cells. Amounts of input viruses were determined by titration in TZM-bl cells to ensure that a virus sample, conferring the highest luciferase activity among all samples used in each experiment, produces a relative light unit (RLU) within an appropriate range (1 × 10^6^ ∼ 8 × 10^6^ RLU). On day 2 post-infection, cells were lysed with 1 × cell culture lysis buffer (Promega Corporation), and were subjected to luciferase assays according to the manufacturer’s instruction. Entry kinetics assay was performed similarly as described previously (Brandenberg et al., 2015). Briefly, virus samples were spin-inoculated into TZM-bl cells at 10°C for 1 hr to ensure virus attachment without entry into cells. Virus-containing media were then replaced with pre-warmed (37°C) fresh media to start virus entry process. At designated time intervals post-inoculation (from 0 to 120 min), a CXCR4 antagonist, AMD3100, was added to culture media at a final concentration of 1 μM to stop further viral entry. On day 2 post-infection, luciferase assays were performed. Entry kinetics were assessed by calculating RLU yielded at each time point post-infection relative to that yielded at 120 min post-infection. To monitor neutralization sensitivity for sCD4 and NAbs, virus samples were pre-incubated with serial twofold dilutions of the reagents at 37°C for 1 h, and were inoculated into TZM-bl cells. Luciferase assays were performed using cell lysates prepared on day 2 post-infection. Neutralization sensitivity was determined by calculating RLU yielded in an appropriate reagent concentration relative to that yielded without the reagent. Reagents used for neutralization assays were as follows, and all anti-HIV-1 gp120 antibodies were obtained from NIH AIDS Reagent Program: sCD4 Human (catalog no. CYT-304; ProSpec-Tany Technogene Ltd.); IgG1 b12 (catalog no. 2640), NIH45-46 G54W (catalog no. 12174), 3BNC117 (catalog no. 12474), VRC01 (catalog no. 12033), and N6 (catalog no. 12968) antibodies that bind to CD4bs; 17b antibody (catalog no. 4091) that recognizes CD4i; CH01 mAb (catalog no. 12561), PG9 (catalog no. 12149), and PGT145 (catalog no. 12703) antibodies that target V1/V2 structure.

### Western Blot Analysis

To determine the expression level of Env proteins, lysates of transfected cells and of virions from transfected cells were prepared as described previously (Fujita et al., 2001, 2008). Lysates, normalized for their Gag-p24 amounts by the HIV-1 p24 antigen ELISA kit (ZeptoMetrix Corporation), were analyzed by western blotting using goat anti-HIV-1 rgp160 (MRC AIDS Directed Program Reagent Project, catalog no. ADP409) and mouse anti-HIV-1 p24 (183-H12-5C) (NIH AIDS Reagent Program, catalog no. 3537) antibodies as previously described (Nomaguchi et al., 2014).

### Flow Cytometry

To determine expression level of cell surface CD4, cells were stained with FITC Mouse Anti-Human CD4 (catalog no. 550628; BD Biosciences) as previously described (Doi et al., 2010; Nomaguchi et al., 2010), and were analyzed by BD FACSVerse Flow Cytometer and BD FACSuite Software (BD Biosciences).

### Molecular Dynamics (MD) Simulations of HIV-1 Env Ectodomain (gp120 and gp41 Ectodomain) With CD4

HIV-1 Env ectodomain bound with CD4 was constructed for ScaVR and its mutant by homology modeling with Molecular Operating Environment (MOE) (Chemical Computing Group). The crystal structure of HIV-1 Env ectodomain (gp120 and gp41 ectodomain) with CD4 at a resolution of 3.7 Å (1 Å = 0.1 nm) (PDB code: 5VN3) (Ozorowski et al., 2017) was used as a modeling template. MD simulations were performed as described previously (Alam et al., 2016; Hikichi et al., 2016; Yokoyama et al., 2016). Briefly, the simulations were done with the pmemd module in the AMBER 16 program package (AMBER 2016, University of California, San Francisco) using the AMBER ff14SB (Maier et al., 2015) and GLYCAM_06j-1 (Kirschner et al., 2008) force field, and the TIP3P water model for simulations of aqueous solutions (Jorgensen et al., 1983). A non-bonded cut-off of 10 Å was used. Bond lengths involving hydrogen were constrained with SHAKE, a constraint algorithm to satisfy a Newtonian motion (Ryckaert et al., 1977), and the time for all MD simulations was set to 2 fs. After heating calculations for 20 ps until 310 K using the NVT ensemble, simulations were executed using the NPT ensemble at 1 atm, at 310 K and in 150 mM NaCl for 500 ns. Free energies of the molecules in solution were calculated from independent all-atom MD trajectories of last 5 ns (100 frames) after 500 ns MD simulations using MMPBSA.py program (Miller et al., 2012) in the AmberTools16 (AMBER 2016, University of California, San Francisco).

## Results

### Three Mutations Accumulated in *env* During *in vitro* Adaptation Experiments Are Able to Individually Increase Viral Replication Potential, Whereas the Virus That Simultaneously Carry the Three Mutations Replicates Most Efficiently in Both Macaque and Human Cells

**FIGURE 1:**
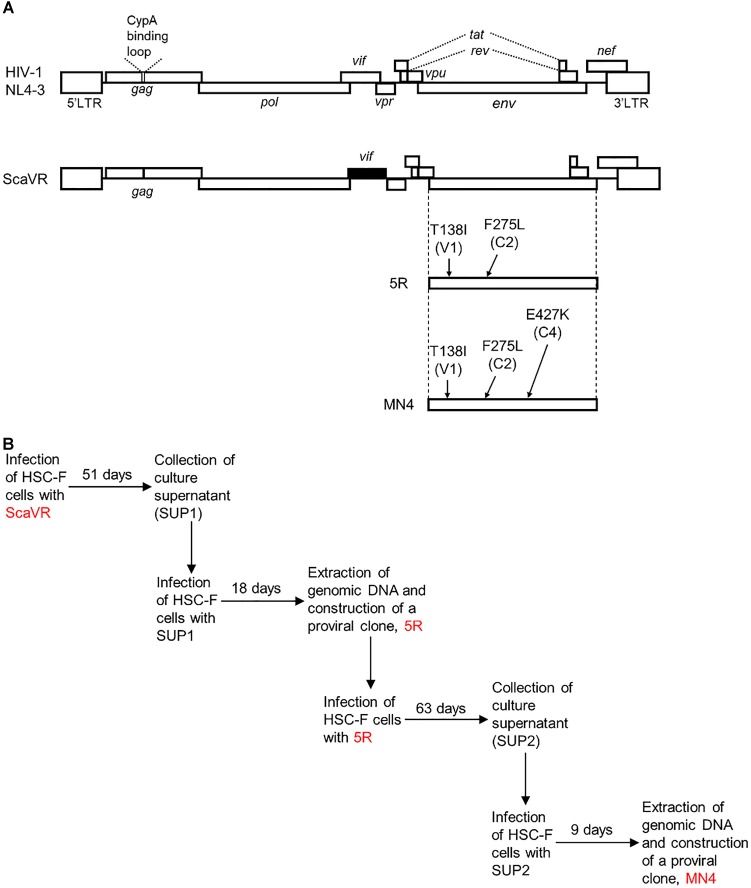
Proviral clones used in this study. **(A)** Genomes of HIV-1_NL4-3_ and its derivative HIV-1mt clone (ScaVR). Sequences from HIV-1_NL4-3_ (GenBank accession number AF324493) and SIVmac_MA239_ (GenBank accession number M33262) are shown by white and black boxes, respectively. Amino acid mutations and their locations (Env domains in parentheses) in Env-gp120 of 5R and MN4 clones are indicated. CypA, cyclophilin A. **(B)** *In vitro* adaptation processes of HIV-1mt clones in HSC-F cells. Details for prolonged cultures of HSC-F cells infected with ScaVR and 5R (Genbank accession number AB266485) have been reported previously (Kamada et al., 2006; Nomaguchi et al., 2013a).

A prototype HIV-1mt clone, ScaVR, was generated by replacing cyclophilin A-binding loop within *gag*-capsid region and entire *vif* gene of a human-tropic HIV-1_NL4-3_ with the corresponding regions of SIVmac_MA239_ (Figure 1A) (Kamada et al., 2006). By adapting ScaVR to replication in HSC-F cells followed by molecular cloning of the adapted viruses emerged, we successfully obtained a better-growing clone designated 5R (Figure 1). Sequence analysis showed that 5R Env-gp120 contains two amino acid alterations, T138I in V1 domain and F275L in C2 domain (HIV-1_NL4-3_ amino acid numbering from start site M), compared to ScaVR (Figure 1) (Kamada et al., 2006). Then, we carried out a next round of virus adaptation in HSC-F cells using 5R, and another proviral clone 5RA with an increased replication ability was finally generated (Nomaguchi et al., 2013a). While several new mutations were noted in the 5RA genome, the only amino acid change in Env-gp120 responsible for viral growth enhancement in HSC-F cells was E427K within C4 domain (Nomaguchi et al., 2013a). We therefore constructed MN4 clone that has above three substitutions (T138I, F275L, and E427K) within distinct domains (V1, C2, and C4, respectively) of Env-gp120 relative to ScaVR Env-gp120 (identical sequence with HIV-1_NL4-3_ Env-gp120) (Figure 1).

**FIGURE 2:**
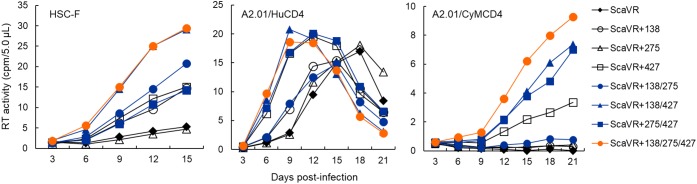
Growth kinetics of ScaVR and its Env-gp120 mutants. Viruses were prepared from 293T cells transfected with proviral clones indicated and inoculated into HSC-F, A2.01/HuCD4, and A2.01/CyMCD4 cells as described in Section “Materials and Methods.” Virus replication was monitored by RT activity released into culture supernatants. Representative data from at least two independent experiments are shown.

While we did not determine previously the effects of T138I and F275L mutations on viral replication potential (Kamada et al., 2006), we showed that the E427K mutation was ineffective in a human cell line MT4/CCR5 (Nomaguchi et al., 2013a). To fully assess the growth-enhancing and species-specific effects conferred by the three mutations, various proviral clones and cell lines were newly generated. We introduced single, double and triple Env-gp120 mutations (T138I, F275L, and/or E427K) into parental clone ScaVR, and constructed seven mutant clones (ScaVR+138, ScaVR+275, ScaVR+427, ScaVR+138/275, ScaVR+138/427, ScaVR+275/427, and ScaVR+138/275/427). Cell lines that stably express human CD4 or CyM CD4 were also generated for comparative replication study from a human cell line A2.01, a CD4-negative variant of CEM T-cell line A3.01 (Folks et al., 1986). A2.01 cells were transfected with either human CD4 or CyM CD4 expression vector, and were selected for CD4 expression by hygromycin B. Resultant human and macaque CD4-positive cells were designated A2.01/HuCD4 and A2.01/CyMCD4, respectively (Figure 2).

Input viruses for infection experiments here were prepared from 293T cells transfected with ScaVR, ScaVR+138, ScaVR+275, ScaVR+427, ScaVR+138/275, ScaVR+138/427, ScaVR+275/427 or ScaVR+138/275/427, and were inoculated into HSC-F, A2.01/HuCD4, and A2.01/CyMCD4 cells. In HSC-F and A2.01/HuCD4 cells, replication-enhancing effects of single, double, and triple mutations in Env-gp120 on viral growth property were readily observed in most cases as shown in Figure 2. While ScaVR+275 and parental ScaVR exhibited similar replication kinetics, ScaVR+138 and ScaVR+427 grew more efficiently than ScaVR. These results have demonstrated the ability and inability of T138I/E427K and F275L mutations, respectively, to clearly promote viral growth in both CyM and human cell lines. Although we found no detectable effect of E427K mutation in human MT4/CCR5 cells previously (Nomaguchi et al., 2013a), this mutation did enhance viral replication in human A2.01/HuCD4 cells used in this study (Figure 2). This is likely to be due to different viral clones and cell lines used in the two studies. In our previous study, viral clones with an intrinsically high replication potential (5R and HIV-1_NL4-3_) and a target cell line with ability to efficiently support viral infection (MT4/CCR5) were dually used, and thus, it is quite conceivable that we were unable to detect a small increase in viral growth. A double mutant ScaVR+138/427 grew more robustly than their corresponding single mutants ScaVR+138 and ScaVR+427, whereas ScaVR+138/275 and ScaVR+275/427 grew similarly with ScaVR+138 and ScaVR+427, respectively. Consistent with the apparent lack of biological effect by F275L mutation described above, a double mutant ScaVR+138/427 and a triple mutant ScaVR+138/275/427 grew similarly and best. In A2.01/CyMCD4 cells, viral replication rates were overall slower than those in the other two cell lines, and thus, virus growth of several clones (ScaVR, ScaVR+138, ScaVR+275, and ScaVR+138/275) was negligible during the observation period (Figure 2). However, growth-enhancing effect of F275L mutation was obvious, since ScaVR+275/427 grew certainly better than ScaVR+427. Moreover, ScaVR+138/275/427 replicated more efficiently in A2.01/CyMCD4 cells than all the other clones tested in Figure 2.

**FIGURE 3:**
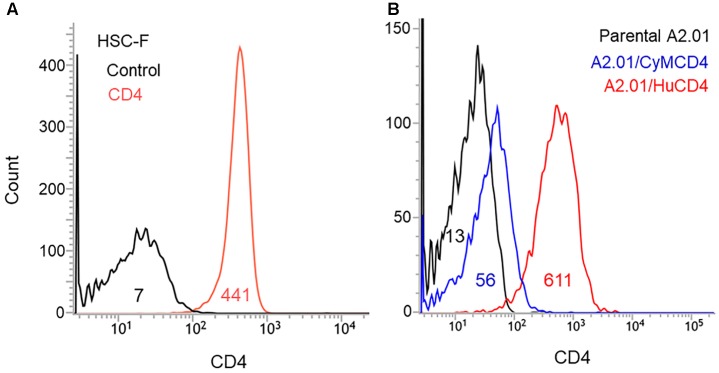
Surface CD4 expression on the cell lines used in this study. Various cell lines were stained with FITC-labeled CD4 antibody, and were subjected to flow cytometry analysis. Numbers in graphs indicate the mean fluorescence intensity of each sample. **(A)** Analysis of CD4-positive HSC-F cells. Negative control is shown by black line. **(B)** Analysis of A2.01 cell line and its derivatives. Parental A2.01 (CD4-negative), A2.01/CyMCD4, and A2.01/HuCD4 cells are shown by black, blue, and red lines, respectively.

The results shown in Figure 2 have indicated that three mutations in Env-gp120 (T138I, E427K, and F275L) can increase viral replication potential. We were interested in distinct mutational effects observed in the three cell lines used. In this regard, we analyzed the surface CD4 expression levels of the cell lines by flow cytometry, since it has been frequently shown that CD4 receptor density on target cells significantly affect Env-mediated viral entry efficiency (Lee et al., 1999; Joseph et al., 2014; Arrildt et al., 2015; Beauparlant et al., 2017). As shown in Figure 3, HSC-F and A2.01/HuCD4 cells expressed a considerably higher level of surface CD4 than negative controls. Of note, surface CD4 level on A2.01/CyMCD4 cells was slightly higher than that on parental A2.01 cells but significantly lower relative to those on the other two cell lines (Figure 3). The F275L-mediated viral growth enhancement of ScaVR+427 and ScaVR+138/427 was observable in A2.01/CyMCD4 cells but not in HSC-F and A2.01/HuCD4 cells (Figure 2). This could be explained by the difference in surface CD4 expression levels on these cell lines. HSC-F cells express a higher level of CyM CD4 than A2.01/CyMCD4 cells (Figure 3). Generally, HIV-1 clones grow more poorly in cells with a low surface CD4 level than in those with a high level. Thus, A2.01/CyMCD4 cells may relatively slow viral replication rates, and may facilitate detection of a subtle increase in viral replication potential rendered by F275L mutation (see results in the A2.01/CyMCD4 panel in Figure 2). In summary, the above described three adaptive mutations individually and cooperatively promote viral replication, and the virus carrying the triple mutations always replicates best both in CyM and human cells.

### Three Adaptive Mutations (T138I, F275L, and E427K) Affect Interaction Between Env-gp120 and CD4

**FIGURE 4:**
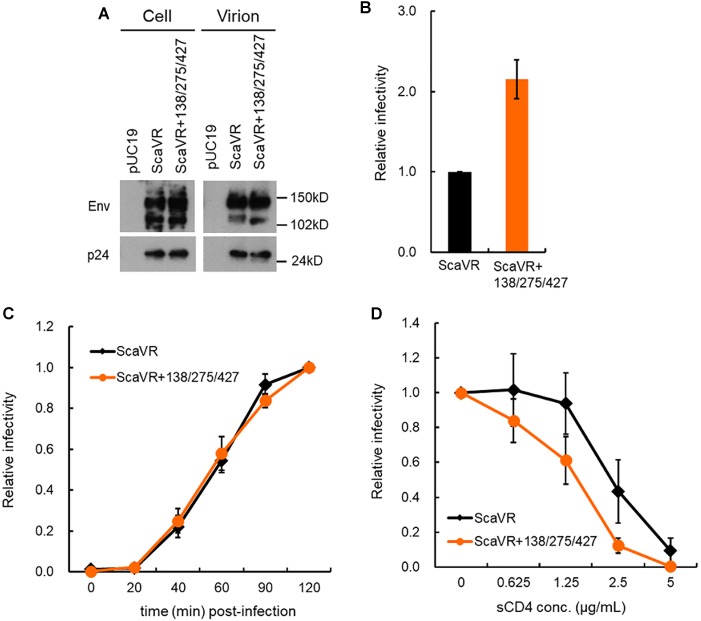
Comparative characterization of ScaVR and ScaVR+138/275/427 viruses. **(A)** Effect of triple mutations in Env on Env expression level. Lysates were prepared from transfected 293T cells as indicated. Samples containing equal Gag-p24 amounts were analyzed by western blotting using anti-HIV rgp160 and anti-HIV-1 p24 antibodies. Representative data from two independent experiment are shown. **(B)** Effect of triple mutations in Env on viral infectivity. Viruses prepared from 293T cells transfected with indicated proviral clones were inoculated into TZM-bl cells. On day 2 post-infection, cells were lysed and used for luciferase assays. Viral infectivity is presented as RLU of ScaVR+3 relative to that of ScaVR. Mean values with standard errors (SE) are shown (*n* = 6). **(C)** Effect of triple mutations in Env on entry efficiency. Virus samples prepared from 293T cells transfected with indicated proviral clones were spin-inoculated into TZM-bl cells at 10°C for 1 h, and were then replaced with pre-warmed fresh media. At indicated time points, AMD3100 was added to culture media at final concentration of 1 μM. On day 2 post-infection, cells were lysed, and subjected to luciferase assays. Relative infectivity is presented by calculating RLU of each virus sample at the indicated time point relative to that of each virus sample at 120 min post-infection. Mean values ± SE are shown (*n* = 4). **(D)** Effect of triple mutations in Env on sCD4 sensitivity. Viruses were prepared from 293T cells transfected with proviral clones indicated, and were incubated with sCD4 at 37°C for 1 h. Virus samples were then inoculated into TZM-bl cells, and on day 2 post-infection, cell lysates were prepared for luciferase assays. Relative infectivity, obtained by calculating RLU of each virus sample incubated with sCD4 relative to that of each virus sample incubated without sCD4, is presented. Mean values ± SE are shown (*n* = 6).

Of the three adaptive mutations, E427K was located in the β20-β21 hairpin which has been suggested to be important for CD4-induced conformational changes (Herschhorn et al., 2017). E427 residue is also located at a CD4 contact site in CD4bs within C4 domain (Pancera et al., 2017). Since these Env mutations enhance viral growth potential, we examined whether the Env expression level in cells and virions differ between ScaVR and its mutant ScaVR+138/275/427 which shows the highest replication ability among the mutants (Figure 2). Cell and virion lysates were prepared from 293T cells transfected with ScaVR or ScaVR+138/275/427, and subjected to western blotting analysis. As is clear in Figure 4A, the two clones were found to express Env at a similar level both in cells and virions. To evaluate the effect of the three adaptive mutations on viral entry process, we first comparatively determined the TZM-bl infectivity of ScaVR and its mutant ScaVR+138/275/427, showing the highest replication ability among mutants (Figure 2). Viruses prepared from 293T cells transfected with ScaVR or ScaVR+138/275/427 were inoculated into TZM-bl cells, and on day 2 post-infection, viral infectivity as judged by luciferase assays was monitored. As expected, viral infectivity of ScaVR+138/275/427 was approximately twofold higher than ScaVR (Figure 4B). We next performed entry kinetics assays to assess viral entry efficiency. Both virus clones exhibited almost the same entry kinetics, strongly suggesting their similar entry efficiency after CD4-binding (Figure 4C). It has been reported that primary HIV-1 viruses can acquire sCD4 sensitivity during an *in vitro* long-term culture, and suggested that increase in sensitivity to sCD4 is resulted from the enhanced interaction of Env-gp120 and CD4 (Pugach et al., 2004; Humes and Overbaugh, 2011; Arrildt et al., 2015). We then carried out sCD4 sensitivity assays by pre-incubating each virus sample (ScaVR and ScaVR+138/275/427) with sCD4 and by measuring viral infectivity in TZM-bl cells. As shown in Figure 4D, the sensitivity of ScaVR+138/275/427 to sCD4 was modestly increased, i.e., the infectivity was modestly decreased, relative to that of ScaVR. These results strongly suggest that the three adaptive mutations augment viral infectivity through enhancement of viral entry process by altering the Env-CD4 interaction.

### Three Adaptive Mutations (T138I, F275L, and E427K) Do Not Affect Viral Sensitivity to Neutralization by CD4bs Antibodies, While Increasing Viral Resistance to Neutralization by CD4i and V1/V2 Antibodies

**FIGURE 5:**
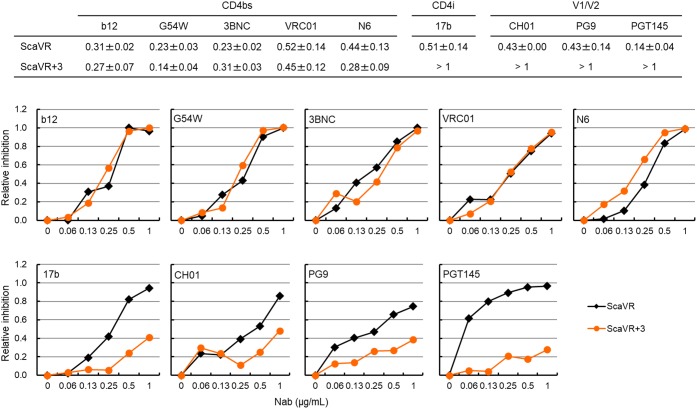
Neutralization sensitivity assays for ScaVR and ScaVR+138/275/427 (ScaVR+3). Viruses were prepared from 293T cells transfected with indicated proviral clones. Virus samples were pre-incubated with each NAb at 37°C for 1 h, and were inoculated into TZM-bl cells. On day 2 post-infection, cells were lysed for luciferase assays. Relative infectivities were calculated by RLU values with indicated concentrations of NAb relative to that without NAb, and then relative inhibition rates (1.0 minus relative infectivity) were determined and are presented. IC_50_ values are indicated in the upper panel. Mean values ± SE are shown (*n* = 6 or more). Representative graph data of neutralization sensitivity assays using NAbs are shown. b12, IgG1 b12; G54W, NIH45-46 G54W; 3BNC, 3BNC117; CH01, CH01 mAb.

*In vitro* adaptation of primary HIV-1 isolates in human T-cell lines and PBMCs has been reported to result in enhancement of their sensitivity to sCD4 and several NAbs (Moore and Ho, 1995; Wrin et al., 1995; Clapham and McKnight, 2002; Pugach et al., 2004). It has been shown that mutations in HIV-1 Env (A204E and G312V), emerged by adapting SHIV in macaque cells, independently promote Env/macaque CD4-mediated viral entry, and that these two mutations influence the sensitivity of SHIVs carrying HIV-1 Env to NAbs (Boyd et al., 2015). Moreover, changes in the sensitivity of HIV-1 strains to NAbs can be linked with alteration in Env trimer conformation (Boyd et al., 2015). Since the three adaptive mutations in this study (T138I, F275L, and E427K) are located in distinct domains (V1, C2, and C4, respectively), and may affect the Env-CD4 interaction as described above (Figure 4), we examined whether the Env mutations change viral sensitivity to various NAbs that target CD4bs [IgG1 b12 (b12), NIH45-46 G54W (G54W), 3BNC117 (3BNC), VRC01, and N6], CD4i (17b), and V1/V2 [CH01 mAb (CH01), PG9, and PGT145]. The CD4bs antibodies [b12, G54W, 3BNC, VRC01, and N6 (Burton et al., 1991; Wu et al., 2010; Diskin et al., 2011; Shingai et al., 2013)] recognize conserved structure formed by Env-gp120 trimer (Benjelloun et al., 2012). The 17b antibody recognizes discontinuous epitope induced by Env conformational changes following CD4 binding, and neutralizes only lab-adapted strains such as HIV-1_NL4-3_ (Thali et al., 1993). The PG9 and PGT145 antibodies target quaternary structure of V1/V2 in Env trimer, and the CH01 antibody recognizes a complex conformation epitope (Walker et al., 2009, 2011; Bonsignori et al., 2011). We performed neutralization sensitivity assays as described for sCD4-sensitivity assays (Figure 4C) using NAbs instead of sCD4. Based on the result demonstrating enhanced sensitivity of ScaVR+138/275/427 to sCD4 (Figure 4C), it was rational to assume that the sensitivity to neutralization by CD4bs antibodies may increase through more exposed CD4bs. However, as is quite clear in Figure 5, the three adaptive mutations did not significantly affect sensitivity of ScaVR+138/275/427 to CD4bs antibodies. Although the three mutations appeared to influence the sensitivity to N6 antibody, IC50 value difference between ScaVR and ScaVR+138/275/427 was not significant (*p* = 0.078 by *t*-test). In contrast, ScaVR+138/275/427 clearly showed enhanced viral resistance to CD4i (17b) and V1/V2 (CH01, PG9, and PGT145) antibodies relative to ScaVR (Figure 5). Totally, while ScaVR carrying HIV-1_NL4-3_ *env* gene was neutralized easily by the CD4i and V1/V2 antibodies, the three adaptive mutations in Env-gp120 allowed the virus to escape from antibodies that recognize epitopes formed by Env trimer. This result thus has suggested that a biologically significant change in Env trimer conformation may be caused by the three mutations.

**FIGURE 6:**
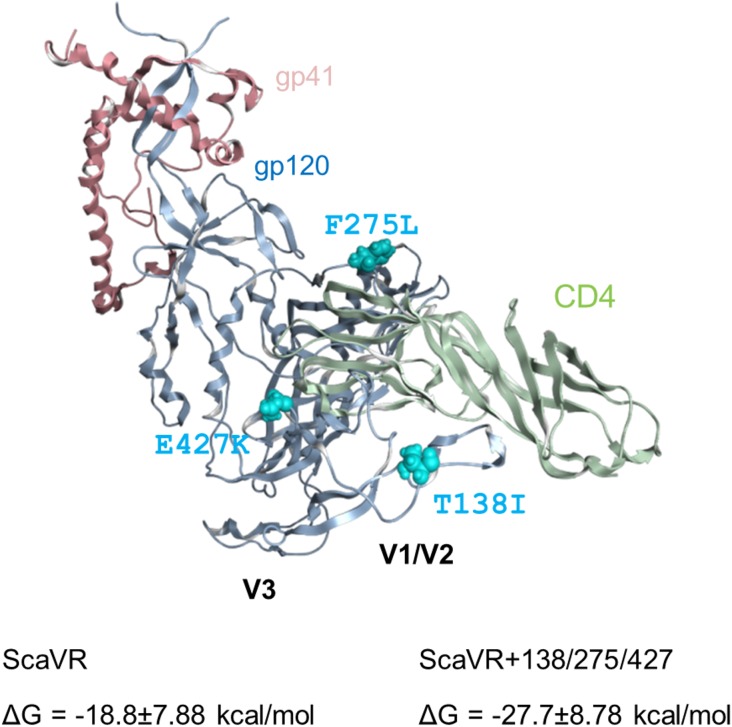
Three-dimensional locations of Env-gp120 mutations in the MD-derived Env ectodomain (gp120 and gp41 ectodomain) bound with soluble CD4. HIV-1 Env ectodomain bound with CD4 was constructed for ScaVR and ScaVR+138/275/427 clones by the homology modeling method using the crystal structure of HIV-1 Env ectodomain with CD4 (PDB code: 5VN3) (Ozorowski et al., 2017). The models were subjected to MD simulations for 500 ns to obtain dynamic structures in solution (see Materials and Methods). Binding energies of the Env proteins to CD4 molecule were calculated using ensembles derived from last 5 ns after 500 ns MD simulations using MMPBSA.py program (Miller et al., 2012) in the AmberTools16 (AMBER 16, University of California, San Francisco). The Env-CD4 complex structure of ScaVR at 100 ns of MD simulation is shown with binding energies of ScaVR and ScaVR+138/275/427 Env proteins to CD4.

Above experimental data suggest that the three adaptive mutations (T138I, F275L, and E427K) induced increase in binding ability of Env to CD4. To address this issue, we conducted *in silico* structural study. Complex model of an Env ectodomain (gp120 and gp41 ectodomain) and a soluble CD4 was constructed for ScaVR and ScaVR+138/275/427 Env clones using the reported complex structure and subjected to the MD simulations to obtain dynamic structures in solution (see Materials and Methods). Subsequently, binding energies of the complexes were calculated using ensembles derived from MD (Miller et al., 2012). Notably, the study has predicted that binding energy of the Env to CD4 in solution will increase by the three adaptive mutations: ΔGs were -18.8 ± 7.88 and -27.7 ± 8.78 kcal/mol for ScaVR and ScaVR+138/275/427 Env-CD4 complexes, respectively (Figure 6). In conclusion, these *in silico* structural data are consistent with the enhanced Env-CD4 interaction experimentally observed for the triple mutant (Figure 4).

## Discussion

In this study, we investigated three adaptive mutations in HIV-1 Env-gp120 (T138I, F275L, and E427K) for their functional and structural characteristics. These mutations spontaneously arose in adaptation processes in macaque cells of HIV-1mt clones derived from HIV-1_NL4-3_. The mutations act against unfavorable circumstances individually and cooperatively to augment viral replication capacity, and the virus that simultaneously carries the three adaptive mutations grew best among clones tested in macaque and human cells (Figure 2). Env-sCD4 interference and entry kinetics assays suggested that the mutations enhance viral replication potential via increase in the interaction of Env-CD4 (Figures 2, 4). These results were supported by MD simulations that predicted increased binding energy of ScaVR+138/275/427 Env to CD4 relative to that of ScaVR Env (Figure 6). Neutralization sensitivity assays have revealed that these mutations can increase resistance to neutralization by CD4i and V1/V2 antibodies, suggesting the change of overall Env-trimer conformation (Figure 5). These results may imply that single-amino acid mutations within V1, C2, and C4 domains cooperatively act on function and structure of HIV-1 Env.

We have selected and summarized several relevant studies on HIV-1 adaptation process (Table 1). *In vitro* adaptation in human T-cell lines and PBMCs of primary HIV-1 isolates appears to commonly lead to increased affinity of their Env proteins for sCD4 and several NAbs (Moore and Ho, 1995; Wrin et al., 1995; Clapham and McKnight, 2002; Pugach et al., 2004). Several SHIVs bearing *env* genes from transmitted/founder viruses or HIV-1 primary isolates exhibit reduced ability to use macaque CD4 as a receptor. Through adaptations in macaque cells or individuals, SHIVs carrying such *env* genes gained a single-amino acid mutation that enhances macaque CD4-mediated entry (Humes and Overbaugh, 2011; Humes et al., 2012; Boyd et al., 2015; Del Prete et al., 2017). However, Env mutations that confer the ability to enhance macaque CD4 utilization were not uniformly distributed among SHIVs (Table 1). Moreover, while adaptive mutations (A204E and G312V) that had emerged during *in vitro* adaptation changed neutralization properties, the other mutation (A281T) that had been acquired during *in vivo* adaptation did not significantly affect neutralization sensitivities to NAbs tested (Boyd et al., 2015; Del Prete et al., 2017). This could probably be due to difference of *env* genes and host environments used for adaptation (Table 1). Neutralization-sensitive viruses (HIV-1/SHIVs), which had become as such during prolonged culture periods, were reported to re-acquire neutralization-resistance through adaptation processes in an experimentally infected macaque or an accidentally infected human (Cheng-Mayer et al., 1999; Beaumont et al., 2001; Si et al., 2001). In this study, we found that a neutralization-sensitive HIV-1_NL4-3_ Env becomes resistant to NAbs’ neutralization by acquiring the three adaptive mutations (T138I, F275L, and E427K) during *in vitro* adaptation, albeit to a lesser degree previously reported (Table 1).

Parental HIV-1mt clone, ScaVR, used in this study was generated from HIV-1_NL4-3_ as a backbone (Kamada et al., 2006). HIV-1_NL4-3_ is categorized into a ‘tier 1’ virus that displays high sensitivity to antibody-mediated neutralization (Munro et al., 2014). A recent study reported that CD4i antibody, 17b, is accessible to some tier 1 Env proteins tested prior to their structural rearrangement induced by CD4 engagement (Boliar et al., 2018). Env on surface of HIV-1_NL4-3_ virions frequently adopts CD4-induced conformation. This can explain the increased sensitivity of HIV-1_NL4-3_ Env to 17b’s neutralization (Munro et al., 2014). Also, in agreement with the results of this study (Figure 5), PG9 and PGT145 antibodies that recognize V1/V2 quaternary epitope has been shown to potently neutralize HIV-1_NL4-3_ (Walker et al., 2009, 2011). Because the three adaptive mutations (T138I, F275, and E427K in V1, C2, and C4 domains, respectively) increased resistance to neutralization by CD4i and V1/V2 antibodies (Figure 5), we speculated that the structural adaptation of Env for increasing affinity for CD4 had simultaneously altered structural property around some Env epitopes. For example, E427K substitution alters the electric charge around the bridging sheet and therefore might alter the molecular interaction with the CD4i antibody. T138I substitution alters hydrophobicity of the V1/V2 portion and therefore might influence the interactions with the V1/V2 antibodies. On the other hand, the three substitutions did not alter viral susceptibility to CD4bs antibodies, indicating that the Env contact sites of the CD4 and CD4bs antibodies are different and that structural properties of the latter were not significantly influenced by the three mutations. Whatever the structural changes are, our study clearly shows that the structural adaptation of Env for increasing viral growth ability under no selective pressures of antibodies does not always result in increase in viral susceptibility to Env antibodies. Molecular understanding of such evolutionary pathways may provide a basis to develop HIV-1 that effectively grows in monkeys.

**Table 1:** Summary of some HIV-1/SHIV adaptation studies.

Name of original clone	Env derived from	Subtype	Tropism	Adapted in	Name of adapted clone	Replication ability	Sensitivity^∗^				Env mutation (domain)^∗∗^	Reference
							sCD4	CD4bs (NAb name)	V1/V2 (NAb name)	CD4i (17b)		
CC1/85	Primary isolate		R5	Human PBMC (19 weeks)	CC con.19	Up in macrophages	Up	up (b12)			I165K and/or D167N, S188del, and N189del (V2)	Pugach et al., 2004
NAB01	Primary isolate	B	R5	Human PBMC (39 weeks) +CD4^low^ targets (18 weeks)	CD4^low^.c21	Decreased infectivity on CD4^high^ target cells		Up (b12), similar (VRC01)	Down (PGT145)	Up	I165K (V2), F317L (V3), T402P, T408N, T412N (V4), G459del, G460del, and N461D (V5)	Beauparlant et al., 2017
					CD4low.c24	similar infectivity on CD4low target cells		up (b12), similar (VRC01)	down (PGT145)	up	I165K and R178K (V2), N197D (C2), F317L (V3), G459del, G460del, and N461D (V5)	
SHIV1054	transmitted/ founder virus	B		Rhesus macaques (92.7, 43.6, and 11 weeks for P1A, P2A, and P3A, respectively)	P1A/P2A/P3A	a small increase in rhesus PBMCs	similar	similar (b12, VRC01)	similar (PG9)		A281T (C2)	Del Prete et al., 2017
HIVAQ23/ SIVVif	Molecular clone Q23-17	A	R5	Immortalized pig-tailed macaque lymphocytes (32 or 35 days)		up in pig-tailed macaque PBMCs	up	similar (VRC01)	down (PG9, PGT145)	up by A204E, similar by G312V	A204E (C2), G312V (V3)	Boyd et al., 2015
ScaVR	molecular clone NL4-3	B	X4	Immortalized CyM lymphocytes (HSC-F cells) (Figure 1)	5R/MN4	CyM PBMCs and individuals	up	similar (b12, G54W, 3BNC, VRC01), a small increase in N6	down (CH01, PG9, PGT145)	down	T138I (V1), F275L (C2), and E427K (C4)	This study


*^∗^In NAb columns, only changes in the sensitivity of the Env proteins to the NAbs used in this study are indicated for easy comparison. ^∗∗^Amino acid numbers are those described in the original articles.*

Env proteins from macrophage-tropic viruses, which can efficiently use CD4 receptor at a lower density, were highly sensitive to sCD4 neutralization compared with those from subject-matched T cell-tropic viruses. This finding indicates that increase in sCD4 sensitivity may imply enhanced interaction of Env-gp120 and CD4 (Arrildt et al., 2015), and can be accounted for by (1) more exposed CD4bs (probably leading to increased sensitivity to CD4bs antibodies), (2) tendency to adopt CD4-induced conformation, or (3) increase in affinity for CD4 (Yen et al., 2014; Arrildt et al., 2015). ScaVR+138/275/427, which has three mutations in the *env* gene from HIV-1_NL4-3_, did not significantly increase sensitivity to five CD4bs antibodies tested relative to parental ScaVR (Figure 5). Although we cannot definitely conclude that the observed sensitivity of ScaVR+138/275/427 to the CD4bs antibodies is unrelated to the small increase in its sensitivity to sCD4 neutralization, it is unlikely that the three mutations cause further exposure of CD4bs on the tier 1 HIV-1_NL4-3_ Env. The triple mutations were also found to enhance resistance of the HIV-1_NL4-3_ Env to a CD4i antibody (Figure 5), which indicates no further shift from its original state (intrinsically highly sensitive state) toward a CD4-induced conformation. Additionally, the entry kinetics of ScaVR+138/275/427 was similar to those of ScaVR, suggesting the three mutations are unlikely to enhance the entry process after CD4-binding (Figure 4B). Furthermore, our MD simulations predict that the three mutations would increase binding affinity of the Env for CD4 in solution (Figure 6). The data are consistent with the reported results that E427 is located at a CD4 contact site in the β20-β21 loop and that T138I is located in the V1 region that supports binding of CD4 (Yokoyama et al., 2016). In total, the increase in sCD4 sensitivity in the Env triple mutant (ScaVR+1385/275/427) is highly likely to be associated with its enhanced affinity for CD4.

Analyses on spontaneous and biologically relevant mutations found in adaptation studies contribute to the identification of mutable sites, where HIV-1 viruses change viral replication potential and NAb sensitivity but maintain their viability. The search and identification of such mutable sites is important as well as determination of epitopes targeted by NAbs, escape mutations from host immune response, and anti-viral drug resistance mutations, etc. Moreover, analysis of changes in function and structure of viral proteins by adaptive mutations serves to establish new way to control viral replication: taking the adaptation ability of viruses to host environments into accounts to act against viruses.

## Author Contributions

ND designed the research, performed the experiments, and discussed the results. MY performed the experiments, discussed the results, and wrote the manuscript. TK performed the experiments and discussed the results. OK discussed the results. HS discussed the results and wrote the manuscript. AA designed the research, discussed the results, and wrote the manuscript. MN designed the research, performed the experiments, discussed the results, and wrote the manuscript.

## Conflict of Interest Statement

The authors declare that the research was conducted in the absence of any commercial or financial relationships that could be construed as a potential conflict of interest.
